# Singapore Grouper Iridovirus ORF75R is a Scaffold Protein Essential for Viral Assembly

**DOI:** 10.1038/srep13151

**Published:** 2015-08-19

**Authors:** Fan Wang, Yang Liu, Yi Zhu, Bich Ngoc Tran, Jinlu Wu, Choy Leong Hew

**Affiliations:** 1 Mechanobiology Institute, National University of Singapore, Singapore; 2 Department of Biological Sciences, National University of Singapore, Singapore

## Abstract

Singapore Grouper Iridovirus (SGIV) is a member of nucleo cytoplasmic large DNA viruses (NCLDV). This paper reports the functional analysis of ORF75R, a major structural protein of SGIV. Immuno fluorescence studies showed that the protein was accumulated in the viral assembly site. Immunogold-labelling indicated that it was localized between the viral capsid shell and DNA core. Knockdown of ORF75R by morpholinos resulted in the reduction of coreshell thickness, the failure of DNA encapsidation, and the low yield of infectious particles. Comparative proteomics further identified the structural proteins affected by ORF75R knockdown. Two-dimensional gel electrophoresis combined with proteomics demonstrated that ORF75R was phosphorylated at multiple sites in SGIV-infected cell lysate and virions, but the vast majority of ORF75R in virions was the dephosphorylated isoform. A kinase assay showed that ORF75R could be phosphorylated *in vitro* by the SGIV structural protein ORF39L. Addition of ATP and Mg^2+^ into purified virions prompted extensive phosphorylation of structural proteins and release of ORF75R from virions. These data suggest that ORF75R is a novel scaffold protein important for viral assembly and DNA encapsidation, but its phosphorylation facilitates virion disassembly. Compared to proteins from other viruses, we found that ORF75R shares common features with herpes simplex virus VP22.

Iridovirids belong to a group of the nucleo cytoplasmic large DNA viruses (NCLDV), infecting a broad range of hosts including invertebrates, amphibians and cold-blooded vertebrates. They have caused huge economic losses in the aquaculture industry and the population decline of some amphibians^1^,^2^,^3^. Their ability to infect the larvae of mosquitoes, a vector for many human diseases, has attracted increasing interest as a potential biological control agent^4^. In order to comprehensively understand these viruses, 17 viral genomes, which represent the members of the five genera in the family *Iridoviridae*, have been sequenced^1^,^5^,^6^,^7^. Structural study has revealed that iridovirid is a typical large icosahedral dsDNA virus that has a similar structure with other dsDNA viruses such as *Paramecium bursaria chlorella* virus^8^. Structure-based phylogenetic tree analysis of major capsid proteins shows that iridoviridae together with other nine virus families including adenoviridae form a cluster harbouring a double jelly-roll motif. This would suggest that these viruses have common ancestors and co-evolved with different hosts^9^. Large DNA viruses devote a significant proportion of their coding capacity to ensure efficient replication and assembly. They have more complex virion structures and replication cycles compared with RNA viruses and ssDNA viruses^10^. To better protect their DNA from environmental damage, large dsDNA viruses have multiple layers^10^. For example, African swine fever virus (ASFV), a species morphologically close to iridovirids, has a central electron-dense nucleoprotein core surrounded by an inner core shell, two inner envelopes, and an outer capsid layer^11^. Mimivirus, another member of NCLDV, has six recognizable layers outside its DNA core^12^. As the virus becomes larger, the packaging process becomes more complicated. Several scaffold proteins are essential for virion structure stability and virion packaging. For example, herpes simplex virus encodes VP22 as a major scaffold protein to help virion stability and the recruitment of several other viral structural proteins^13^.

ORF75R is a major structural component in mature SGIV virion^14^. We have previously identified that it was a lipid-binding protein and phosphorylated at multiple sites^15^. Knockdown of ORF18R, another abundant structural protein of SGIV, could affect ORF75R phosphorylation and virion packaging^16^. Here we demonstrate that ORF75R is a scaffold protein important for viral packaging.

## Results

### ORF75R accumulates in the viral assembly site (VAS)

**Figure 1:**
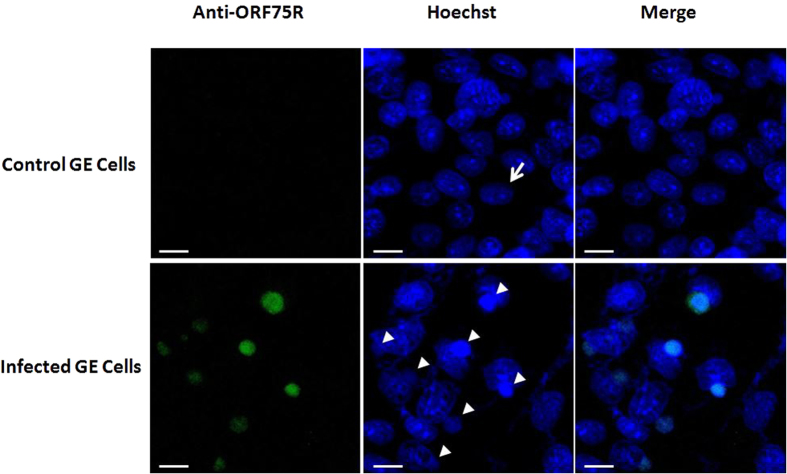
ORF75R is highly accumulated in viral assembly site. The monolayers were fixed at 36 h.p.i., permeabilized with chilled methanol and processed using the indicated combination of primary antibody and Alex Fluor 594-conjugated secondary antibody. Cellular dsDNA was stained with Hoechst 33342. The target protein is represented in green, dsDNA is represented in blue. Scale bars, 10 μm. The uninfected cell nucleus is indicated by arrow heads and viral assembly site by arrows.

The localization of ORF75R during SGIV infection was first visualized via fluorescence staining. Unlike the control cells, in which the cellular chromatin was observed as unevenly distributed blue staining (Fig. 1, arrow) in the nucleus, virus-infected cells contained small dense blue dots close to the nuclei (Fig. 1, triangles), which was VAS as previously described^17^. Moreover, ORF75R immunostaining (Fig. 1, green) merged perfectly with the blue dots (Fig. 1, triangles), which indicated that ORF75R was highly accumulated in the VAS.

### ORF75R is essential for viral infection and virion packaging

**Figure 2:**
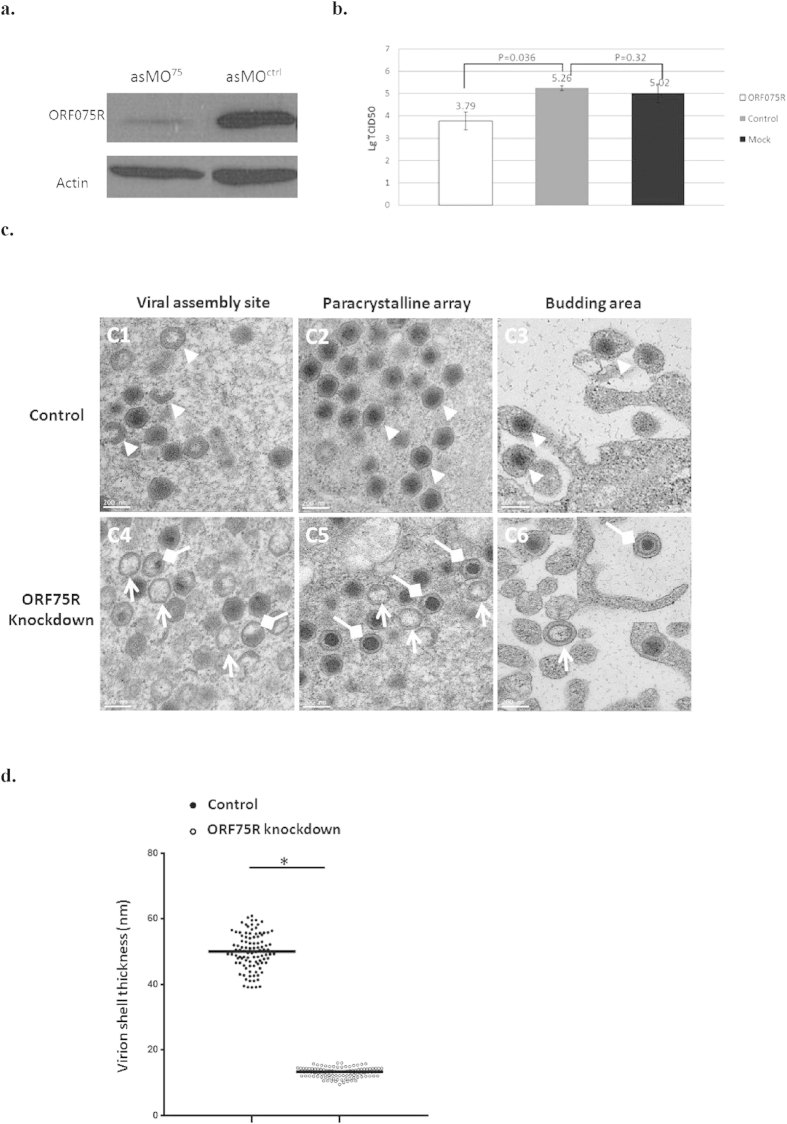
ORF75R is critical for virus infectivity and virionpackaging. (**A**) ORF75R Knockdown examined by Western blotting. GE cells transfected with asMO^ctrl^ and asMO^75^ were infected with SGIV (MOI = 1) at 24 hrs post-transfection. Total cell lysates were collected and analyzed at 48 h.p.i. (**B**) TCID_50_ assay of ORF75R-knockdown viruses. GE cells transfected with asMO^ctrl^, asMO^75^ and mock transfection were infected with SGIV (MOI = 1) at 24 hrs post-transfection. Samples were collected at 48 h.p.i. The variation of the viral titers was evaluated by the student’s t-test. (**C**) Effect of ORF075R knockdown on SGIV assembly. GE cells transfected with asMO^ctrl^ (C1, C2, C3) or asMO^18^ (C4, C5, C6) were infected with SGIV (MOI = 5) at 24 hrs post-transfection. At 48 h.p.i., the cells were fixed and prepared for electron microscopy. C1 and C4 represent the viral assembly site (VAS) region; C2 and C5 represent the paracrystalline region; C3 and C6 represent the budding area. The pictures shown are representatives of at least 20 different virus-infected cells. Viruses in control cells are indicated by arrow heads and defective viruses in knockdown cells by arrows and diamond arrows. Scale bars, 200 nm. (**D**) Thickness of wild-type and ORF75R knockdown virion shell. Each symbol represents a measured shell thickness of each partially packaged virion; small horizontal lines indicate the mean. *P < *0.01* (Student’s t-test).

To understand the role of ORF75R in SGIV infection, ORF75R expression was inhibited by antisense morpholino. The expression of ORF75R was drastically inhibited in the presence of asMO^**75**^ (Fig. 2a). A TCID_50_ assay indicated that the yield of infectious particles was also significantly reduced in the presence of asMO^**75**^ (Fig. 2b). This result has two possible explanations, namely either fewer virions were generated or defective virions without infectivity were produced.

To further examine the cause of the knockdown effect, the same MOI-infected cells (including both asMO^**ctrl**^**-** and asMO^**75**^**-**delivered grouper cells) were fixed and compared via TEM. As shown in Fig. 2c, no significant differences of virion numbers were found in the VAS, the paracrystalline array region or the budding area between the asMO^**ctrl**^- and asMO^**75**^-delivered cells. However, the depletion of ORF75R caused the generation of defective virions in these subcellular compartments. The concentric multi-layered structure (core shell) was observed in the VAS of asMO^**ctrl**^-delivered cells (Fig. 2c1, triangle). However, the multi-layered structure became thinner in asMO^**75**^-delivered cells when compared with control cells (Fig. 2c4, arrows). Further observation revealed another type of defective virions in VAS which contained an improperly packaged DNA core (Fig. 2c4, diamond arrows). The heterogeneous viral morphologies were probably caused by the incomplete deletion of ORF75R in asMO^**75**^-delivered cells. The virions without ORF75R would have an assembled capsid shell but no DNA core, whereas virions with trace amount of ORF75R would have an abnormal DNA core. The virions with significant amount of ORF75R would have a normal DNA core. Hence both normal and defective virions were observed in asMO^**75**^-delivered cells. Defective virions without a DNA core (Fig. 2c5, arrows) or with distorted core (Fig. 2c5, diamond arrows) were also observed in the paracrystalline array region surrounding the VAS in asMO^**75**^-delivered cells. Only mature virions with a substantial core were observed in the paracrystalline array region in control cells (Fig. 2c2, triangle). Observation of the budding area found that normal enveloped budding viruses were present in control cells (Fig. 2c3, triangle), while enveloped viruses without core (Fig. 2c6, arrow head) or with distorted core (Fig. 2c6, diamond arrow) were present in asMO^**75**^-delivered cells. To better quantified this phenotype, one hundred ORF75R knockdown virions and one hundred wild type virions have been picked up for the measurement of core shell thickness. The results have been shown as a scatter plot and the ORF75R knockdown virion had much thinner shell than wild type virion statistically (Fig. 2d).

Our data clearly indicated that ORF75R played an important role in viral DNA encapsidation but might not be involved in capsid shell assembly and virion budding process. The generation of defective virions was the major reason for the low yield of infectious particles.

### ORF75R is localized beneath virion capsid

To further determine the location of ORF75R in SGIV virion, an immunogold labeling experiment was carried out. The gold particle was observed between the virion capsid shell and DNA core (Fig. s1). Although cryo-EM observation of *Chilo iridescent* virus (CIV)^8^ has revealed detailed information of the viral capsid shell, the internal structure of iridovirids was less understood. This experimental data indicated that ORF75R was an important component of the multiple concentric layered structures (core shell), which were observed in SGIV and CIV under both TEM and cryo-EM^8^,^16^ with different electro densities.

### Lipid bilayer beneath virion capsid is not affected by ORF75R knockdown

**Figure 3:**
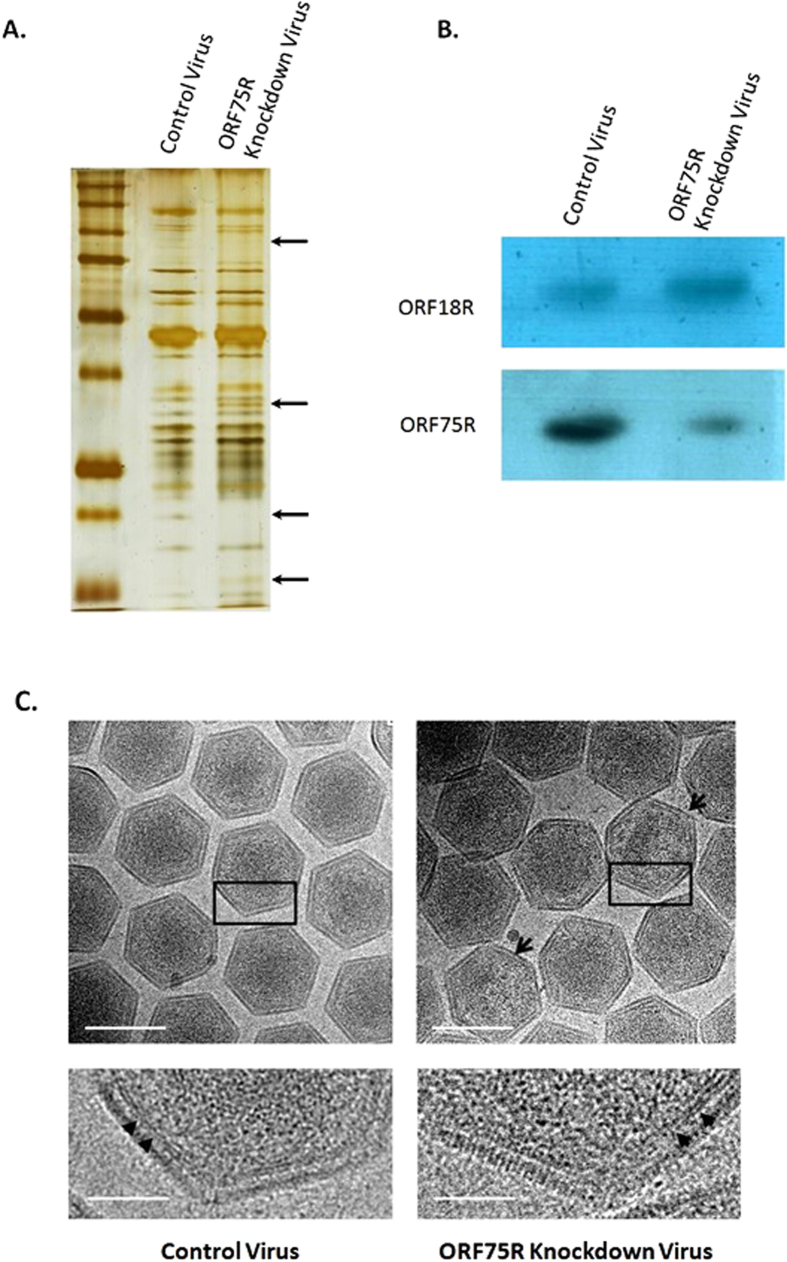
Lipid bilayer beneath virion capsid is not affected by ORF75R knockdown. (**A**) Silver staining of a protein gel from purified control virions and ORF75R-knockdown virions. Purified control virions (1 μg) and ORF75R-knockdown virions (1 μg) were analyzed by SDS-PAGE. The prominent differences were indicated by arrows. (**B**) ORF75R knockdown examined by Western blotting. ORF18R and ORF75R were detected by Western analysis using anti-ORF75R and anti-ORF18R. (**C**). Cryo-EM observation of control viruses and ORF75R-knockdown viruses. Purified control viruses and ORF75R-knockdown viruses were observed under cryo-EM. The lower panels are magnified from the boxed area of the upper panels. The pictures shown are representative of at least 100 virions. The virions without cores are indicated by arrows and the inner lipid layers are indicated by arrow heads. Scale bars for upper panels, 200 nm; Scale bars for lower panels, 50 nm.

Previous cryo-EM study of CIV revealed that it had an inner membrane beneath the capsid shell^8^. Because ORF75R was a lipid binding protein^15^ and was located under the capsid shell (Fig. s1), it was important to find out whether the virion inner lipid membrane was affected by ORF75R knockdown. Purified virions from ORF75R knockdown sample were prepared. The quality of purified knockdown virions was examined by silver staining (Fig. 3a) and Western Blot (Fig. 3b). The protein composition of virions was affected by ORF75R knockdown (Fig. 3a, arrows). ORF75R was reduced significantly based on Western blotting analysis (Fig. 3b). The purified virions were also processed and subjected to cryo-EM observation. Virions without a DNA core (Fig. 3c, arrow head) were observed in ORF75R knockdown sample as anticipated, which was consistent with our previous TEM observations of ORF75R knockdown in ultra-thin section of SGIV-infected cells. Enlarged images showed that the lipid bilayer beneath capsid shell was present in ORF75R-knockdown virions (Fig. 3c, triangle). Both the capsid shell and inner membrane could form with reduced-ORF75R.

### Comparative quantification of virion structural proteins between control virus and ORF75R-knockdown virus

**Figure 4:**
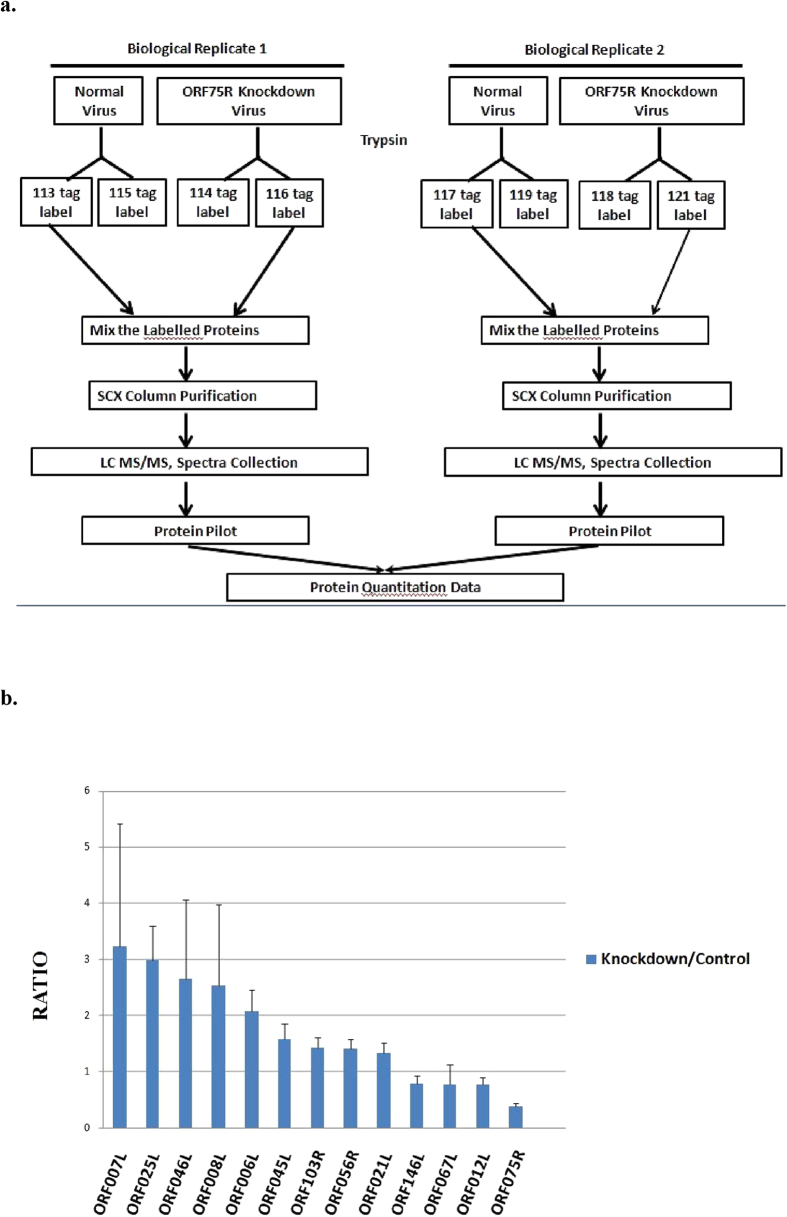
Quantitative comparison of virion structural proteins between control viruses and ORF75R-knockdown viruses. (**A**) Experimental flowchart of iTRAQ. (**B**) Significantly altered viral structural proteins after ORF75R knockdown. The ratio is calculated from the protein amount in knockdown viruses over its amount in control viruses. All the values are the average of four replicates with standard deviation.

To investigate whether the depletion of ORF75R would affect the recruitment of other viral proteins in virion assembly, two independently purified control and ORF75R-knockdown virus samples were prepared for iTRAQ experiments. Each sample was equally divided into two parts and labeled with different tags as replicates. To ensure the reliability of data, only the proteins that had more than two peptides identified in all samples were compared. In total, 54 viral proteins were identified from normal and ORF75R-knockdown virions. Nine of them were upregulated (ratio > 1.3) and four were down regulated (ratio < 0.8) after ORF75R knockdown (Fig. 4b). ORF75R was the most down regulated protein (ratio = 0.38) while major capsid protein was not significantly changed (ratio = 1.09) (Table s1). These data were consistent with previous cryo-EM observations that SGIV capsid shell was not affected by ORF75R knockdown (Fig. 3c). Among the significantly changed proteins, ORF067L (ratio = 0.78) is a core gene encoded protein. ORF007L (3.24), ORF25L (2.99) ORF046L (2.66), ORF008L (2.53), ORF006R (2.09), ORF045L (1.58), ORF103R (1.43), ORF056R (1.41), ORF21L (1.34), ORF146L (0.79) and ORF012L (0.78) are conserved in *Ranavirus*^5^. Several other viral genome-associated proteins such as ORF146L (helicase, 0.79) and ORF67L (Deoxynucleoside kinase, 0.78) were down regulated with ORF75R knockdown, which was consistent with previous cryo-EM observations that the virion DNA core was abnormal upon ORF75R knockdown (Fig. 3c). These data further validated the role of ORF75R as an important scaffold protein that is involved in the recruitment of other viral structural proteins during virion assembly. Among the upregulated proteins, five of the nine upregulated proteins (ORF007L, 008L, 045L, 046L, 056R) were grouped as collagen-like proteins^5^. ORF008L was previously identified as a matrix protein in the viral core^18^. The relatively high amounts of these proteins in ORF075-knockdown virions may result from two possibilities: depletion of ORF075 provides more space in virions for other core proteins to compensate for the core structure; depletion of ORF075 allows these proteins to be more easily detected by mass spectrometry.

### ORF75R is phosphorylated *in vitro* and *in vivo*

**Figure 5:**
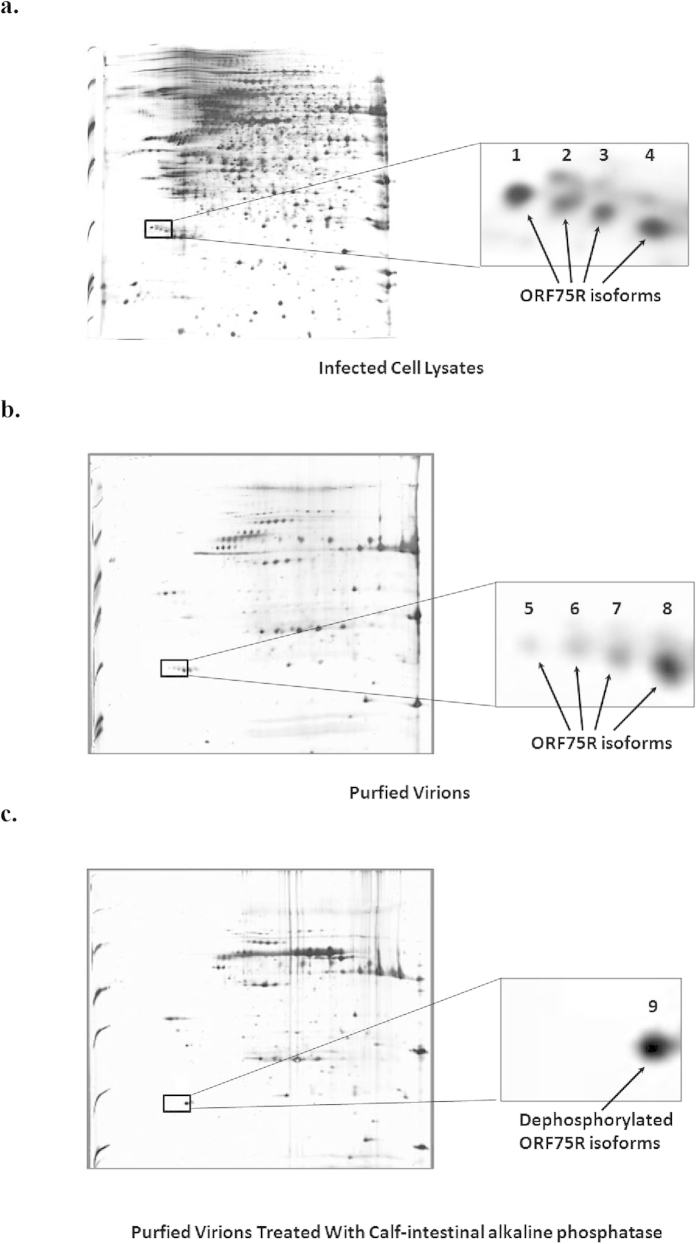
ORF75R is phosphorylated at multiple sites and dephosphorylated ORF75R is the majority in mature virions. (**A**) 2DE analyses of SGIV-infected cell lysate. GE cells were infected with SGIV (MOI = 1). 150 μg cell lysate was collected at 72 h.p.i. and analyzed by 2DE. (**B**) 2DE analyses of purified SGIV virions. 10 μg purified virions were analyzed by 2DE. (**C**) 2DE analyses of CIP-treated SGIV virions. 10 μg purified virions were incubated with 200 u CIP in the PBS with 0.5% NP40 for 30 min at 30 °C before 2DE. Right panels were enlarged from boxed area of left panels.

Previously we have identified that ORF75R had multiple isoforms and it has been assumed that these were phosphorylated isoforms^16^. To confirm this, purified SGIV virions and CIP-treated SGIV virions were compared and analyzed via two dimensional SDS-PAGE (Fig. 5b,c). The four spots (Fig. 5b: Spot5, 6, 7, 8) identified in purified virions merged into one single spot (Fig. 5c, Spot9) in CIP-treated virions. The four spots observed on the 2D gel have earlier been identified as ORF75R via mass spectrometry^16^ and we confirmed that Spot9 was also ORF75R (Table s2). We further identified three serine/threoninephosphorylated peptides from Spot1 while no phosphorylated peptides were found in Spot9 (Fig. s2). These data confirmed that Spot9 was dephosphorylated and ORF75R was phosphorylated *in vivo.*

**Figure 6:**
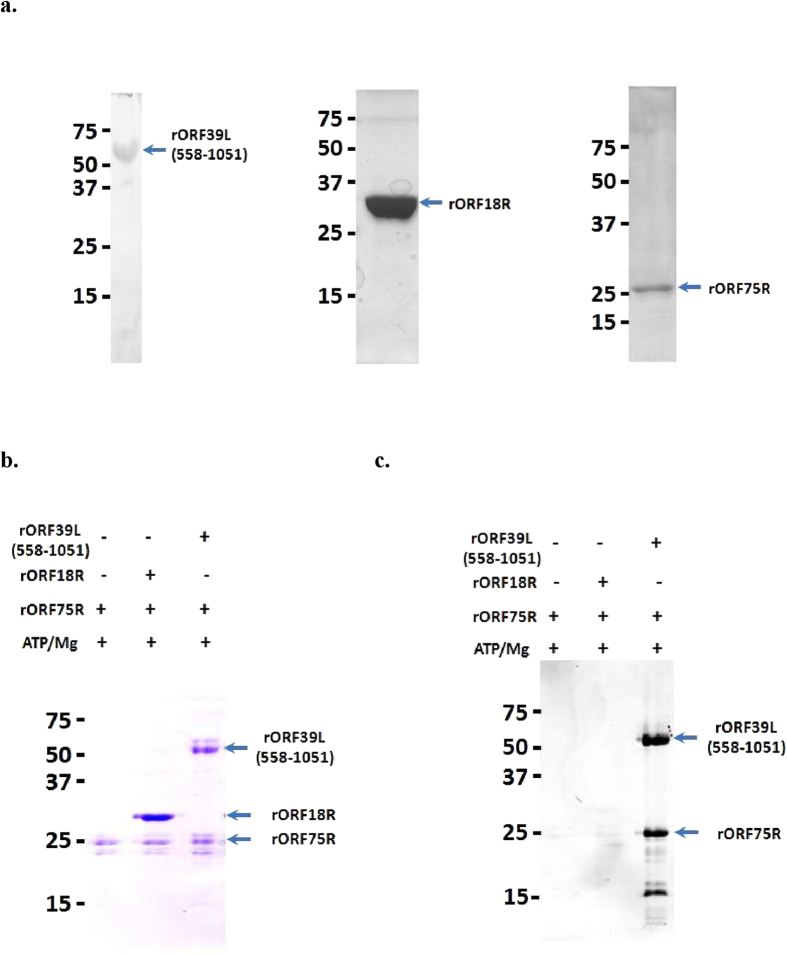
ORF75R is phosphorylated by ORF39L *in vitro*. (**A**) Purified ORF75R, ORF18R and truncated ORF39L (558–1051). Purified rORF075R (1 μg) was phosphorylated by addition of (1) 1 mM ATP, 10 mM MgCl_2_, (2) 1 mM ATP, 10 mM MgCl_2_, 1 μg purified rORF018R and (3) 1 mM ATP, 10 mM MgCl_2_, 1 μg purified rORF039R (558–1051) in 10 μl final vol. The reaction mixtures were incubated at 25 °C for 10 min and the reaction was stopped by addition of 5 X SDS loading dye (250 mM Tris-HCl pH 7.0, 50% glycerol, 0.5% Bromophenol Blue, 20% SDS, 200 mM DTT). The samples were loaded for SDS-PAGE and the gel was stained with (**B**) Coomassie Blue and (**C**) ProQ Diamond (Invitrogen).

Comparative genomic analyses have indicated that SGIV *ORF39L* and *ORF18R* are conserved genes in family Iridoviridae^5^. Both of these two proteins contained conserved kinase domains and were highly abundant structural proteins^14^. Thus ORF75R, ORF18R and truncated ORF39L containing the putative kinase domain (558–1051) were purified for kinase activity assay (Fig. 6a). ProQ® diamond staining indicated that ORF75R could be phosphorylated by ORF39L kinase domain but not by ORF18R *in vitro* (Fig. 6c).

### The majority of ORF075 in mature virions is dephosphorylated ORF75R

To elucidate the function of ORF75R phosphorylation, SGIV-infected cell lysate and purified SGIV virions were compared via 2D gel electrophoresis (Fig. 5a,b). The ratios of these four ORF75R isoforms were different between infected cell lysate and purified virions. Phosphorylated ORF75R (Spot1) was the most abundant isoform in SGIV-infected cell lysate while dephosphorylated ORF75R (Spot8) was the most abundant isoform in purified SGIV virions (Fig. 5). These data indicated that the dephosphorylated isoform of ORF75R accumulated in mature virions while the phosphorylated isoforms of ORF75R were more prominent in infected cells.

### ORF75R is released out of mature virions after phosphorylation

**Figure 7:**
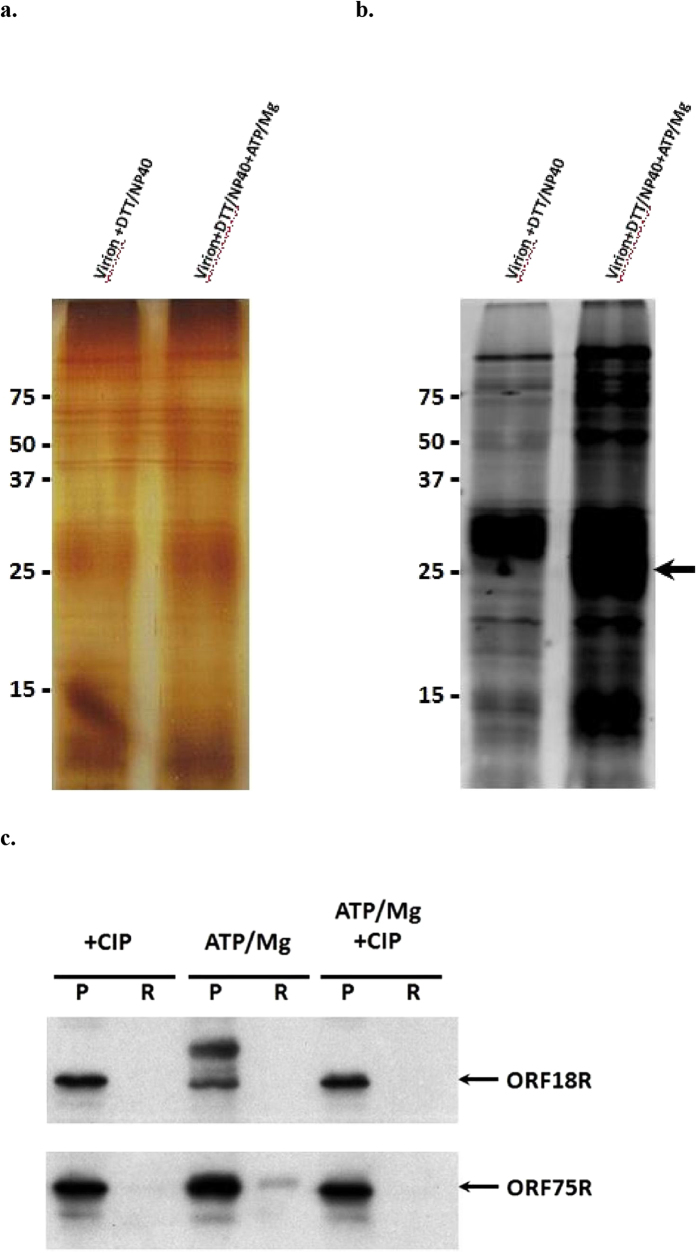
Viral structural proteins were phosphorylated in the presence of ATP and Mg^2+^. Purified virion (30 μg) was disrupted in 25 mM Tris-HCl (pH 8.0) buffer, 10 mM DTT, 0.5% NP40 at 30 °C for 30 min, and proteins were phosphorylated by addition of (1) Buffer only and (2) 1 mM ATP, 10 mM MgCl_2_ in 200 μl final vol. and incubation at 30 °C for 15 min. The reaction was stopped by addition of 5 X SDS loading dye (250 mM Tris-HCl pH 7.0, 50% glycerol, 0.5% Bromophenol Blue, 20% SDS, 200 mM DTT). For each lane certain treated virions (10 μg) were loaded on 12% acrylamide gel and stained with (**A**) silver staining and (**B**) ProQ Diamond (Invitrogen). Arrows indicate aheavily phosphorylated region. For dissociation assay (**C**), purified virions (10 μg) were incubated with (1) 200 u CIP, (2) 1 mM ATP, 10 mM MgCl_2_ and (3) 200 u CIP, 1 mM ATP, 10 mM MgCl_2_ in 20 μl of PBS and 0.5% NP40 for 4 hrs at 30 °C. The treated samples were analyzed by Western Blot. P, pelleted material. R, released material.

To determine the significance of this unique distribution of phosphorylation, a functional comparison between SGIV ORF75R and other viral scaffold proteins were performed. We found that VP22, a major scaffold protein from herpes simplex virus, shared common features with SGIV ORF75R. VP22 could be phosphorylated by UL13, the viral gene encoded kinase, and dephosphorylated isoforms of VP22 accumulated in mature virions^19^. Moreover, phosphorylation of mature virion components, including VP22, promoted dissociation of the herpes simplex virus^20^. Based on the similarities between HSV VP22 and SGIV ORF75R, a virion phosphorylation assay was performed and the samples were analyzed by SDS-PAGE and ProQ® diamond staining (Fig. 7a and Fig. 7b). Multiple viral structural proteins were phosphorylated with the addition of Mg^2+^ and ATP (Fig. 7b). A 25 kDa region on SDS-PAGE was heavily phosphorylated (Fig. 7b, arrow), which suggested that ORF75R might be phosphorylated in purified virions. A further sucrose gradient assay revealed that the slow-mobility form of ORF75R (phosphorylated ORF75R) was released into the soluble fraction from purified virions upon addition of ATP and Mg^2+^ while ORF75R could not be released in the presence of Calf-intestinal alkaline phosphatase (CIP) (Fig. 7c). ORF18R also had a slow-mobility isoform upon addition of ATP and Mg^2+^ but no ORF18R was released into the soluble fraction, which might be explained by the strong association of ORF18R with other viral structural proteins. Due to the fact that ORF75R was one of the major structural proteins and could be phosphorylated by another major structural protein ORF39L *in vitro*, its phosphorylation and release were correlated with SGIV virion dissociation.

## Discussion

In this report, two important questions in iridovirid biology have been answered by studying SGIV: 1. Iridovirid contains scaffold proteins which are commonly identified in other large dsDNA viruses^21^,^22^,^23^. 2. Phosphorylation and dephosphorylation of certain viral structural proteins are involved in iridovirid assembly and disassembly.

Cryo-EM image reconstruction has provided detailed information about viral capsid shell structure and organization for iridovirid^8^. However, observation of the detailed viral inner structure has been problematic due to technical limitations. For example, mimivirus, another member of the NCLDV family, contains multiple layers between capsid and DNA core as observed by different electron microscope techniques^24^. However, the detailed protein compositions and function of these multiple layers were largely unknown. Here our data showed that ORF75R was located between virus capsid shell and DNA core. It was not only a key scaffold protein for viral assembly, but could also be used as a tool to analyze virus inner structure. Scaffolds are known to interact and/or bind with multiple partners, tethering them into complexes. For large dsDNA virus, scaffold proteins were commonly used to assist virion assembly and viral DNA packaging. For example, VP22 is a major scaffold protein for herpes simplex virus^21^, D13 is a Double-Barrel scaffold protein of Poxvirus^22^ and L425 is an icosahedral capsid scaffold protein for mimivirus^23^. Iridovirid is also a member of the large dsDNA virus family. Our present functional studies of ORF75R demonstrated that Iridovirid also has at least one such scaffold protein. The lack of this protein could cause abnormal recruitment of other viral structural proteins and distorted virus assembly.

Phosphorylation plays diverse roles in different biological processes. Parasitic viruses utilize phosphorylation for assembly, replication and immune escape^25^. A comparative genomics study has showed that iridovirids contain common serine/threoninekinases^5^. To date, no functional study has been performed about the function of phosphorylation in iridovirids. We show that ORF75R, a major viral structural component, has multiple phosphorylation isoforms in the infected cell lysate and purified virions. The accumulation of ORF75R dephosphorylated isoform in purified virionis of interest. Here, phosphorylation increased ORF75R solubility and facilitated its release from purified virions, which suggest that virion unpackaging and dissociation was related with phosphorylation. Recently people have found that iridovirids entered into the host cells via clathrin-mediated endocytosis in a pH-dependent manner^26^,^27^. Thus what our discoveries have revealed a potential picture that phosphorylation triggered dissociation might be an important event after iridovirid entry. Future study will be focused on whether iridovirid phosphorylation and disassembly are interdependent *in vivo*.

Recently, structural virology has provided evidence that different viruses that infect hosts from bacteria to human might share a common ancestor^28^. Three viral lineages (PRD1-like, HK97-like and BTV-like) have been annotated based on the structural comparison of the viral capsid proteins^29^ and the family Iridoviridae belongs to the PRD1-like lineage^28^. Here, we did functional comparisons between different large dsDNA viruses for the novel iridovirid scaffold protein ORF75R. Some scaffold proteins in the PRD1-like lineage share certain functional similarities with ORF75R, such as L1 52/55k, a packaging scaffold protein in adenoviruses, which could be phosphorylated, cleaved and is involved in virion maturation^30^,^31^. However, the most similar scaffold protein to ORF75R was identified as VP22, from herpes simplex virus, a member of the HK97-like lineage (Table 1). VP22 was initially localized beneath the procapsid and was expelled from the capsid with DNA packaging^32^. Its dephosphorylated isoform was accumulated in the mature viral tegument and phosphorylation could induce its release from mature virions and promote virion dissociation^19^,^20^. Although ORF75R was localized beneath the capsid shell, its dephosphorylated isoform was also accumulated in the mature virion and could be released upon phosphorylation. Such a unique feature is unlikely to have been inherited randomly. Instead, ORF75R from SGIV and VP22 from herpes simplex virus probably share certain evolutionary links although iridovirid and herpes virus belong to two different lineages (PRD1-like and HK97-like, respectively). Although it is controversial whether the major groups of large dsDNA viruses (NCLDV, herpes virus, and baculo viruses) evolved from a common ancestral virus, comparative studies have revealed that some core functional proteins are commonly shared in these three major groups of large dsDNA viruses. For example, a core DNA replication apparatus containing a DNA polymerase, DNA helicase, primase, and PCNA-like DNA clamp is present in herpes viruses, baculo viruses, and the NCLDVs^33^. Here our data provide an additional evidence to support the idea that these viruses descended from a small group of ancestral dsDNA viruses and converged to some extent due to inter-viral exchanges of certain advantageous genes. Further studies on iridovirid life cycle would provide more evidence to understand the evolutionary history of complex large dsDNA viruses.

## Methods

### Cells and viruses

Grouper embryonic (GE) cell line was isolated from the brown-spotted grouper (*Epinephelustauvina*)^34^. Eagle’s minimum essential medium containing 10% fetal bovine serum, 0.116 M NaCl, 100 IU of penicillin G/ml and 100 μl of streptomycin sulfate/ml was used to culture the GE cells at 27 °C. Singapore Grouper Iridovirus (SGIV) was isolated from diseased grouper^35^. A monolayer of GE cells in fresh EMEM culture medium had to reach at least 80% confluence before infection with SGIV. Amplified SGIV in cell culturewas harvested, aliquoted and stored at −80 °C as seeds^14^.

### Antibodies

The purified recombinant ORF18R and ORF075R proteins were used to produce the rabbit anti-ORF18R and anti-ORF075R polyclonal antibodies. The monoclonal antibody (MAb) to actin was from Chemicon. Horseradish peroxidase-conjugated donkey anti-mouse and donkey anti-rabbit polyclonal antibodies were from Pharmacia. The anti -His tag monoclonal antibody was from Abcam. Goat anti-rabbit gold particles (25 nm) and goat anti-mouse gold particles (15 nm) were from Electron Microscopy Sciences. Anti-rabbit Alexa Fluor 488-conjugated antibody was from Molecular Probes (Life Technologies).

### Immunofluorescence (IF) assay

GE cells were cultured to 90% confluence on Lab-Tek^®^ Chambered Coverglass (NalgeNunc), infected with SGIV at a Multiplicity of Infection (MOI) of 1. Cells were fixed at 36 hours post transfection (h.p.i.) with 3% paraformaldehyde, permeabilized with 100% methanol, and then incubated with anti-ORF075R antibody (1:500) for 1 hr, washed for 3 × 10 min in Tris-Buffered Saline and Tween 20 (TBST). Cells were incubated in anti-rabbit Alexa Fluor 488-conjugated antibody for 1 hr, washed for 3 × 10 min in TBST. dsDNA were stained with Hoechst (Life Technologies). Fluorescence was detected with a Zeiss confocal microscope.

### Anti-sense morpholino (asMO) design and transfection

AsMO design was based on the full SGIV sequenced genome (GenBank Accession Number_AY521625) and predicted ORF075R sequences^14^. Moreover, the sequences were screened with BLAST (http://www.ncbi.nlm.nih.gov/BLAST/) against the whole SGIV database to preclude any unintentional gene-silencing effects. All asMOs were synthesized and purified by GeneTools. The sequences of asMO^**ctrl**^ and asMO^**75**^ were CCTCTTACCTCAGTTACAATTTATA and CTCCGAAAATATCGTCGATATCCAT, respectively. AsMOs were delivered as described before^16^. SGIV was inoculated into the transfected cells at 40 hrs post transfection. Infected GE cells were harvested at 48 h.p.i. for downstream analyses or at different time courses for transmission electron microscopy and Tissue Culture Infectious Dose 50 (TCID_50_) test.

### TCID_50_ test

The asMOs transfected GE cells were infected with SGIV at 40 hrs post transfection with an MOI of 1. The asMO^ctrl^ and asMO^75^-delivered cells were harvested, homogenized and pelleted at 48 h.p.i. The supernatant was diluted from 10^−2^ to 10^−9^ and used to infect GE cells with eight repetitions per dilution to perform the TCID_50_ assay. The viral titers were calculated using the Spearman-Karber method^36^.

### Transmission electron microscopy (TEM) and Cryo electron microscopy (Cryo-EM)

The GE cells were harvested and fixed with 2.5% glutaraldehyde, 2% paraformaldehyde in PBS (pH 7.4) overnight. Then the samples were post-fixed with 1% osmium tetroxide for 3 hrs, followed by dehydration in an increasing ethanol series of 50%, 75% and 100% (twice). The samples were embedded using the Spurr kit (Sigma), sliced into ultrathin sections (70–90 μm) and stained with 2% uranyl acetate, 1% lead citrate. The ultrathin sections were viewed under the JOEL JEM 2010F electron microscope. For Cryo-EM, Quantifoil grids were freshly glow discharged at 10 mA at 40 s. 4.5 μl of virus was place on a grid. Grids with sample were plunged freeze in liquid ethane^37^ and viewed under Titan krios 300 KV. The thickness of viral core shell was measured by Image J according to software menu.

### SDS-PAGE and Western blotting

SDS-PAGE, silver staining and Western blotting were carried out as described previously^16^. Pro-Q® diamond phospho protein gel stain (Life Technologies) was carried out following manufacturer’s instructions. For Western blotting, proteins were electro phoretically transferred to poly vinylidenedi fluoride (PVDF) membranes. Western blots were developed using horseradish peroxidase-conjugated secondary antibody (Pharmacia) and the Pierce ECL western blotting substrate according to the manufacturers’ instruction. Nonspecific binding was minimized by addition of 0.2% Tween20 to wash buffers.

### Phosphorylation Assay

For *in vitro* assay, purified rORF075R (1 μg) was phosphorylated by the addition of 1 mM ATP, 10 mM MgCl_2_; 1 mM ATP, 10 mM MgCl_2_, 1 μg purified rORF018R; 1 mM ATP, 10 mM MgCl_2_, 1 μg purified rORF039R (558–1051) in 10 μl final volume. The reaction mixtures were incubated at 25 °C for 10 min and loaded for SDS-PAGE separation. For the *in vivo* assay, purified SGIV (100 μg proteins) was disrupted in 25 mM Tris-HCl (pH 8.0) buffer, 10 mM DTT, 0.5% NP40 at 30 °C for 30 min, and proteins were phosphorylated by addition of buffer only or 0.2 mM ATP, 10 mM MgCl_2_ in 200 μl final volume and incubation at 30 °C for 15 min. The gel was stained with ProQ® diamond staining buffer according to the manufacturer’s instruction and the ProQ®diamond stained gel was scanned with a Typhoon 9200 imager (Ex: 580 nm, Em: 620 nm).

### Virion Dissociation Assay

Purified virions were incubated in 20 μl of PBS and 0.5% NP40 for 4 hrs at 30 °C in the presence or absence of 1 mM MgCl_2_ and 1 mM ATP. The mixture was then layered onto 200 μl of 30% (wt/vol) sucrose in PBS in a micro centrifuge tube and centrifuged at 20000 *g* for 30 min. The 20 μl of supernatant above the interface containing released, fully soluble proteins was carefully collected. The pelleted materials containing insoluble viral proteins and DNA were also collected. In a parallel experiment, 200 U of calf intestinal phosphatase (CIP; NEB) was incorporated into the incubation mixture.

## Additional Information

**How to cite this article**: Wang, F. *et al.* Singapore Grouper Iridovirus ORF75R is a Scaffold Protein Essential for Viral Assembly. *Sci. Rep.* **5**, 13151; doi: 10.1038/srep13151 (2015).

## Supplementary Material

Supplementary Information

## Figures and Tables

**Table 1:** Functional Comparison between SGIV ORF75R and HSV VP22.

	SGIV ORF75R	HSV VP22
Abundance in mature virion	Comparable with major capsid protein^14^	Comparable with major capsid protein^13^
Localization in mature virion	Between capsid and DNA core (Fig. s1)	Beneath the procapsid^38^ and a viral tegument protein in mature virion^13^
Phosphorylation	Phosphorylated by another viral structural protein ORF39L (Fig. 6)	Phosphorylated by another viral structural protein UL13^19^
Localization of dephosphorylated isoform	Accumulated in mature virion (Fig. 5)	Accumulated in mature virion^19^
Phosphorylation function	Promote ORF75R releasing (Fig. 7)	Promote virion dissociation^20^
Loss of function study	Generation of less infectious particles and incorporate less ORF146L and ORF67L (Fig. 2 and Fig. 4)	Release less infectious particles and incorporate less ICP0, gD and gE-gI into virion^39^,^40^
