# Supplementary material for: Singapore Grouper Iridovirus ORF75R is a Scaffold Protein Essential for Viral Assembly
Source: Sci Rep. 2015 Aug 19;5:13151. doi: 10.1038/srep13151 (PMC4541339; doi:10.1038/srep13151)
Supplement: Supplementary Information [file srep13151-s1.pdf]

Title: Singapore Grouper Iridovirus ORF75R is a Scaffold Protein Essential for Viral Assembly

Authors: Fan Wang[1], Yang Liu[1], Yi Zhu[2], Bich Ngoc Tran[2], Jinlu Wu[2*] and Choy Leong Hew[1,2*]

***Corresponding author:**

Jinlu Wu, Department of Biological Sciences, National University of Singapore, Singapore; e-mail: dbswjl@nus.edu.sg, Phone:(65) 65168476, Fax: (65) 67792486.

Choy Leong Hew, Mechanobiology Institute, National University of Singapore, Singapore, Department of Biological Sciences, National University of Singapore, Singapore; e-mail: dbshewcl@nus.edu.sg, Phone:(65) 65167658, Fax: (65) 67792486.

Supplemental Materials and Methods:

Virus purification

The SGIV infected GE cells and growth medium were harvested at 72 h.p.i. and centrifuged at 12,000 × *g* for 30 min at 4°C. The resulting pellet was resuspended with the culture medium and ultrasonicated. The suspension containing the lysate, viral particles and cellular debris were then centrifuged at 4,000 × *g* for 20 min at 4°C. The supernatant was layered onto a cushion of 35% sucrose and centrifuged at 210,000 × *g* for 1 h at 4°C. The pellet was resuspended with the TNE buffer (100 mM Tris; 2.0 M NaCl; 10 mM EDTA; pH 7.4) and overlaid with 30%, 40%, 50% and 60% (m/v) sucrose gradients and centrifuged at 210,000 × *g* for another 1 h at 4°C Viral bands, present between 40% and 50% sucrose, were aspirated, sonicated briefly, and reloaded onto iodixanol gradients (10%, 20%, 30%, and 40%) and centrifuged for at 350,000 × *g* for 16 hrs at 4°C. Virus particles were suspended at the boundary layer between 30% and 40%. This layer was individually aspirated and spun down at 100,000 × *g*. The virus particles were stored at -80°C.

Immunogold Labelling

GE cells were cultured to 80% confluence as a mono-layer and infected with SGIV at a MOI of 3. Infected GE cells were harvested at 24 h.p.i., fixed and cryo-sectioned using the Tokuyasu method [1] with modifications. Cells in the culture medium were fixed with a double-strength fixative of 4% paraformaldehyde (PFM) and 0.4% glutaraldehyde (GA)

in PBS, pH 7.4 for 15 min (volume of that fixative equal to the medium). The medium-fixative mixture was replaced with a fresh single-strength fixative (2% PFM and 0.2% GA in PBS pH 7.4). After 2h fixation at room temperature (RT), cells were harvested using a cell scraper, and centrifuged for 1 min at 1500 rpm. The cell pellet was washed 3 times with PBS, 5 min each, then suspended in 12% wt/vol gelatin in PBS at 37°C for 10 min, centrifuged again and placed on ice for 15 min. A block of cells in gelatin was cut into small cubes (~1 mm^3), immersed in cold 2.3M sucrose and rotated overnight. A small cube of cells was trimmed and cut at -80 °C using a Leica UCT Ultramicrotome and Diatom cryo Diamond knife. Ribbons of cryo sections of ~ 90 nm thickness were transferred to formvar carbon grids (on ice) using a drop of a mixture containing 2 parts of 2.3M sucrose: 1 part of 2% methylcellulose. Grids with thawed sections were incubated in 0.12% glycerin in PBS for 15 min, blocked with 1% BSA in PBS for 30 min and washed 3 times with PBS, 5 min each. Sections were incubated in primary antibodies for 1h (anti ORF075 Ab or anti His-tag Ab, 50× dilution) and then washed 3 times with PBS, 5 min each. Secondary gold antibodies were added and incubated for 1h (goat anti rabbit gold, EMS-25115 or goat anti mouse gold, EMS-25132, 40× dilution) and then washed 6 times with PBS, 5 min each. After that, grids were fixed with 1% GA in PBS for 5 min, then washed 4 times with water, 5 min each. Finally, grids were incubated with a mixture of 1 part of 1% UA: 9 parts of 2% methylcellulose, on ice for 5 min. Grids were dragged on filter paper to remove the excess liquid and air dried before TEM viewing.

Isobaric Tags for Relative and Absolute Quantitation (iTRAQ) Analyses of Control Viruses and ORF75R-Knockdown Viruses

Purified control virions (100 μg) and ORF75R-knockdown virions (100 μg) were dissolved in 10μl urea (10 M) separately at RT. Each sample was incubated with 2 μl tris-(2-carboxyethyl) phosphine (TCEP, 100 mM stock solution) for 1h, and then 2 μl methyl methane-thiosulfonate (MMTS, 200 mM stock solution) was added into each sample for another 10min at RT. Following reduction and alkylation, trypsin (12.5 ng/μl, Promega) was added and incubated at 37°C overnight. The digested peptides from control virion and ORF75R knockdown virion were equally divided into two parts separately for iTRAQ labeling. iTRAQ labeling was performed using the iTRAQ Reagent kit (AB SCIEX, Foster City, CA, USA) following manufacturer's instructions. To remove interfering substances, the pooled iTRAQ-labelled peptides was subjected to strong cation exchange chromatography (SCX) using the iTRAQ Methods Development Kit (AB SCIEX, Foster City, CA, USA). The bound peptides were eluted with 5% ammonium hydroxide (NH_4OH) in 30% methanol. They was desalted using a Sep-Pak C_{18} cartridge (Waters, Milford, MA), dried and then reconsitituted with 100 μl of diluents (98% water, 2% acetonitrile, 0.05% formic acid). The samples were analyzed on a TripleTOF 5600 system (AB SCIEX, Foster City, CA) with Protein Pilot System.

Table S1. Comparative proteomics analysis of control virus and ORF75R-knockdown virus

a. Biological replicate one

%Cov(95)	Name	Peptides (95%)	114:113	PVal 114:113	115:113	PVal 115:113	116:113	PVal 116:113
93.30	ORF072R	727	1.17	0.13	1.02	0.68	1.14	0.13
69.55	ORF039L	284	1.13	0.09	1.05	0.28	1.15	0.09
51.86	ORF012L	259	0.93	0.28	1.16	0.01	0.93	0.29
71.91	ORF026R	155	0.76	0.00	0.90	0.10	0.79	0.01
56.60	ORF060R	94	1.32	0.00	1.04	0.34	1.32	0.00
66.79	ORF069L	142	0.82	0.04	1.07	0.33	0.79	0.00
47.69	ORF057L	92	1.33	0.00	1.04	0.50	1.37	0.00
52.53	ORF078L	74	1.21	0.02	1.00	0.99	1.21	0.01
59.29	ORF088L	66	1.32	0.01	1.06	0.35	1.31	0.00
53.62	ORF090L	79	1.36	0.00	1.09	0.17	1.49	0.00
65.56	ORF156L	51	1.62	0.00	1.08	0.49	1.53	0.00
52.56	ORF089L	51	1.16	0.11	1.09	0.31	1.16	0.13
60.74	ORF137R	42	1.03	0.71	1.00	0.99	1.04	0.66
53.07	ORF084L	41	1.57	0.00	1.13	0.03	1.64	0.00
88.84	ORF045L	79	1.34	0.08	1.04	0.77	1.36	0.04
90.00	ORF008L	62	1.27	0.07	1.02	0.84	1.19	0.21
36.54	ORF093L	51	1.13	0.18	1.04	0.52	1.15	0.14
78.23	ORF046L	71	1.47	0.05	1.13	0.35	1.48	0.02
71.57	ORF101R	44	1.81	0.00	0.87	0.08	1.85	0.00
79.17	ORF055R	91	1.02	0.86	0.99	0.93	1.01	0.91
57.89	ORF018R	75	1.17	0.25	1.10	0.46	1.22	0.21
31.48	ORF016L	32	2.34	0.00	1.02	0.87	2.42	0.00
62.35	ORF038L	68	0.85	0.37	0.81	0.29	0.80	0.32
53.14	ORF162L	48	0.70	0.00	1.14	0.14	0.71	0.00
58.44	ORF009L	59	0.53	0.33	0.64	0.38	0.62	0.18
41.37	ORF025L	23	3.73	0.00	1.09	0.30	3.67	0.00
50.00	ORF019R	46	1.11	0.59	0.93	0.44	1.22	0.16
53.93	ORF075R	27	0.35	0.00	1.12	0.34	0.32	0.00
81.71	ORF056R	19	1.29	0.12	1.03	0.84	1.32	0.17
23.24	ORF043R	14	0.49	0.00	1.11	0.37	0.51	0.00
37.35	ORF022L	39	1.40	0.14	0.90	0.20	1.50	0.09
33.59	ORF006R	16	1.83	0.00	1.01	0.91	1.81	0.00
65.58	ORF086R	16	0.77	0.33	1.10	0.68	0.76	0.24
62.30	ORF067L	18	0.48	0.00	1.10	0.40	0.42	0.00

58.99	ORF021L	16	1.56	0.00	1.19	0.03	1.66	0.00
47.52	ORF020L	18	1.21	0.26	1.34	0.11	1.25	0.15
56.85	ORF059L	21	1.02	0.82	0.92	0.51	0.98	0.91
42.16	ORF061R	19	0.88	0.33	1.04	0.64	0.90	0.47
49.19	ORF007L	27	1.45	0.21	1.31	0.16	1.47	0.07
22.53	ORF146L	18	0.76	0.11	1.20	0.07	0.74	0.02
30.58	ORF152R	13	1.17	0.16	1.17	0.05	1.07	0.36
9.71	ORF052L	7	0.97	0.79	1.06	0.37	1.13	0.38
35.29	ORF081L	10	0.99	0.91	1.03	0.75	1.09	0.43
49.34	ORF070R	14	1.28	0.16	1.19	0.54	1.22	0.10
37.14	ORF122L	11	1.14	0.44	1.13	0.23	1.10	0.52
18.58	ORF134L	5	0.85	0.13	1.24	0.07	0.80	0.04
63.64	ORF102L	13	0.87	0.61	1.41	0.07	0.98	0.88
12.01	ORF150L	6	0.96	0.87	1.11	0.47	0.99	0.95
42.17	ORF119R	9	1.05	0.86	0.90	0.67	1.04	0.83
18.04	ORF111R	5	0.52	0.08	0.79	0.42	0.54	0.06
35.59	ORF015L	12	1.25	0.51	1.04	0.86	1.06	0.78
15.99	ORF118R	3	0.77	0.06	1.26	0.24	0.92	0.79
22.52	ORF082L	4	0.65	0.07	0.93	0.45	0.73	0.40
9.91	ORF155R	4	0.92	0.84	1.08	0.78	1.17	0.88
48.54	ORF139R	7	1.13	0.23	1.06	0.65	1.11	0.56
6.12	ORF064R	4	0.64	0.32	1.11	0.49	0.73	0.20
35.87	ORF125R	7	0.41	0.04	1.18	0.52	0.45	0.14
14.61	ORF098R	5	1.78	0.01	1.24	0.09	1.69	0.05
11.63	ORF087R	4	0.54	0.10	1.24	0.46	0.81	0.36
24.34	ORF115R	3	0.64	0.29	1.02	0.91	0.95	0.82
19.59	ORF103R	3	1.49	0.30	1.17	0.37	1.37	0.20
28.37	ORF014L	4	1.20	0.64	1.04	0.87	1.35	0.39
2.08	ORF128R	2	1.08	0.57	1.51	0.15	0.97	0.79
6.40	ORF147L	2	0.48	0.08	0.80	0.45	0.58	0.38
7.27	ORF132R	2	1.15	0.51	1.27	0.26	1.18	0.27

b. Biological replicate two

%Cov(95)	Name	Peptides (95%)	118:117	PVal 118:117	119:117	PVal 119:117	121:117	PVal 121:117
95.68	ORF072R	886	1.05	0.72	1.05	0.61	1.02	0.87
72.79	ORF039L	266	0.64	0.00	0.81	0.01	0.71	0.00
50.2	ORF012L	181	0.59	0.00	0.80	0.01	0.64	0.00
75.97	ORF026R	168	1.03	0.63	1.11	0.17	1.01	0.86
44.78	ORF057L	82	1.12	0.13	0.98	0.78	1.13	0.00
52.58	ORF060R	80	1.03	0.69	0.94	0.43	1.15	0.00
60.04	ORF069L	126	0.84	0.01	0.88	0.11	0.92	0.11
75.56	ORF156L	89	0.65	0.00	0.84	0.16	0.71	0.00
56.57	ORF090L	112	0.65	0.00	0.90	0.50	0.67	0.00
51.39	ORF078L	46	1.04	0.58	0.92	0.26	1.09	0.10
61.46	ORF088L	57	1.07	0.46	0.85	0.07	1.16	0.09
83.39	ORF101R	57	0.78	0.03	0.94	0.54	0.77	0.00
57.63	ORF016L	62	0.90	0.43	0.86	0.18	1.02	0.76
58.44	ORF009L	85	1.14	0.70	1.66	0.32	0.98	0.96
65.61	ORF018R	65	0.62	0.00	0.79	0.11	0.69	0.00
42.17	ORF022L	66	0.93	0.63	0.88	0.48	1.04	0.66
52.05	ORF089L	44	0.79	0.03	0.87	0.25	0.83	0.00
58.82	ORF038L	74	1.30	0.34	1.43	0.15	1.11	0.60
50	ORF019R	43	0.84	0.12	0.90	0.27	0.89	0.11
85.12	ORF045L	53	1.96	0.00	1.16	0.19	1.97	0.00
54.45	ORF137R	29	0.92	0.19	0.88	0.25	0.99	0.76
90	ORF008L	45	4.53	0.00	1.57	0.06	4.86	0.00
84.96	ORF056R	28	1.50	0.01	1.14	0.32	1.76	0.00
48.27	ORF084L	33	1.06	0.78	0.99	0.98	1.05	0.72
66.94	ORF046L	45	4.32	0.00	1.48	0.00	5.08	0.00
72.08	ORF055R	52	1.49	0.01	0.97	0.84	1.69	0.00
37.65	ORF025L	23	2.38	0.00	1.15	0.12	2.82	0.00
36.13	ORF162L	19	1.35	0.00	0.90	0.27	1.56	0.00
29.63	ORF093L	31	0.76	0.09	1.07	0.66	0.69	0.00
43.14	ORF061R	19	0.83	0.17	0.98	0.91	0.86	0.13
50	ORF075R	21	0.44	0.00	0.94	0.70	0.42	0.00
52.44	ORF007L	47	5.79	0.00	1.65	0.04	7.13	0.00
29.94	ORF146L	18	0.72	0.04	0.79	0.08	0.85	0.05
54.61	ORF070R	15	0.80	0.40	0.80	0.29	0.90	0.31
11.47	ORF052L	8	1.47	0.00	1.01	0.86	1.49	0.00
23.54	ORF152R	11	1.15	0.15	1.12	0.27	1.17	0.04
27.8	ORF006R	8	2.55	0.01	1.33	0.60	2.85	0.00

34.78	ORF020L	8	1.87	0.06	1.25	0.05	2.55	0.02
43.46	ORF067L	7	1.11	0.54	1.05	0.80	1.22	0.11
46.58	ORF059L	11	0.83	0.23	0.89	0.48	0.96	0.65
71.43	ORF102L	19	0.96	0.81	0.78	0.17	1.19	0.29
55.4	ORF021L	21	1.16	0.32	0.95	0.68	1.18	0.06
54.37	ORF139R	7	1.09	0.49	0.99	0.97	1.08	0.58
71.08	ORF119R	10	1.60	0.06	1.29	0.19	1.32	0.25
38.71	ORF049L	5	0.97	0.91	0.98	0.93	0.83	0.09
37.5	ORF135L	4	1.07	0.49	0.94	0.78	0.97	0.76
8.741	ORF064R	4	1.55	0.01	1.06	0.76	1.66	0.03
34.76	ORF081L	11	1.25	0.22	1.01	0.96	1.19	0.25
23.81	ORF122L	4	0.92	0.49	0.88	0.19	0.94	0.46
23.22	ORF134L	6	2.02	0.02	1.17	0.48	1.38	0.29
20.34	ORF015L	5	0.85	0.42	1.10	0.58	0.64	0.17
11.81	ORF150L	4	1.19	0.44	1.31	0.47	1.09	0.85
11.99	ORF098R	4	0.92	0.70	1.15	0.78	1.23	0.62
15.29	ORF111R	3	1.92	0.40	1.53	0.30	1.71	0.50
12.85	ORF118R	3	2.09	0.22	1.85	0.10	1.39	0.42
26.37	ORF048L	2	1.25	0.62	1.13	0.86	1.07	0.79
42.27	ORF103R	2	1.72	0.13	1.28	0.56	1.70	0.20
8.49	ORF004L	2	0.91	0.51	0.98	0.93	1.09	0.58
7.24	ORF115R	2	0.78	0.49	0.73	0.62	0.90	0.66

Table S2. Mass Spectrometry Identified ORF75R for Spot 9.

Spot No.	Protein Name	NCBI Accession No.	MW (Da)	pI	No. of matched peaks	Mascot Protein Score	Total Ion score	Sequence Coverage (%)
9	ORF075R [SGIV]	gi|56692712	19949	4.5	4	80	62	8

Figure S1

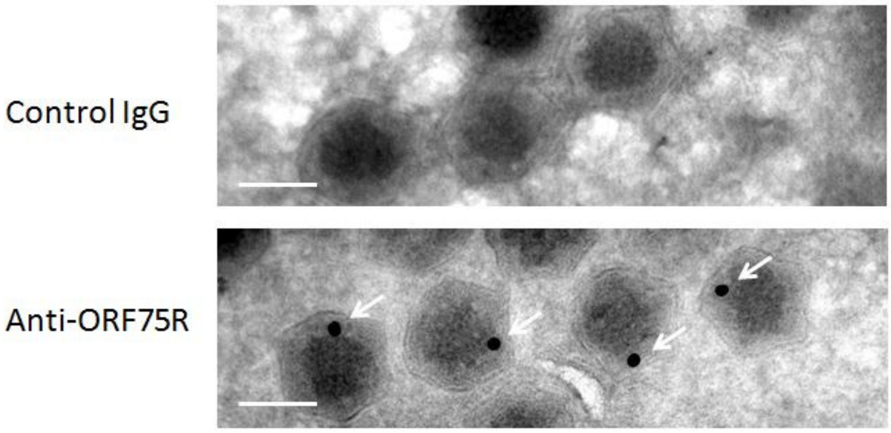

Figure S1. ORF75R is Localized beneath Virion Capsid Shell. GE cells were infected with SGIV (MOI=3) and harvested at 24 h.p.i., fixed and cryo-sectioned using the Tokuyasu method. The section was incubated with anti-his serum or anti-ORF75R serum. 10nm gold particles were indicated by arrows. Scale bars, 100nm.

Reference

1. Slot, J.W. & Geuze, H.J. Cryosectioning and immunolabeling. *Nature protocols* **2**, 2480-2491 (2007).

Figure S2. Identified Phospho Peptide Spectra for Spot 1

MDIDDIFGDLGDMTDDETGEETDDDFDENVEGGDYAEFPDDK**ENLSAIPLHE**
RPLYNPKILAHGELLPNERILDFSILETAATRKIATEDYSDKELNLLPLVALVDR
YTQLK**ALDSALRSEFR**VVLDLPAVADMHLRNVAKLILYQR**GKVTVPYAAYN**
LLRYAAKRGLINPPGHGKST

Fragment sequence	Modification	MW	z	MW/z
ENLSAIPLHER		1277.67	2	639.84
	Phospho(S)@4	1357.62	2	679.83
ALDSALRSEFR		1263.65	2	632.83
	Phospho(S)@4	1343.61	2	672.82
GKVTVPYAAYNLLR		1563.84	2	782.94
	Phospho(T)@4	1643.83	2	822.93

Spot 1 identified spectrum:

ENLSpAIPLHER

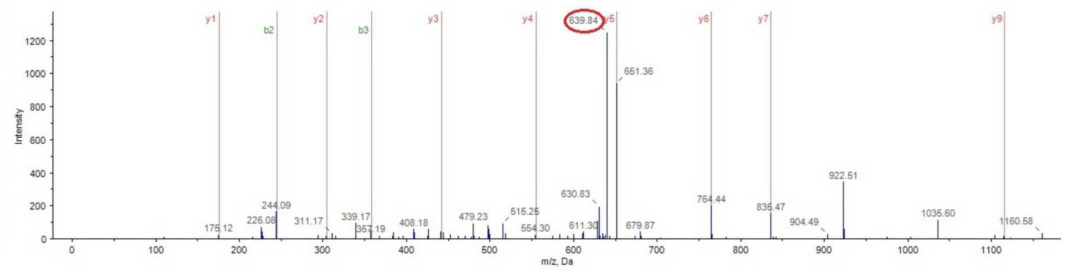

Modification: phosphorylation at S residue **ALDSpALRSEFR**

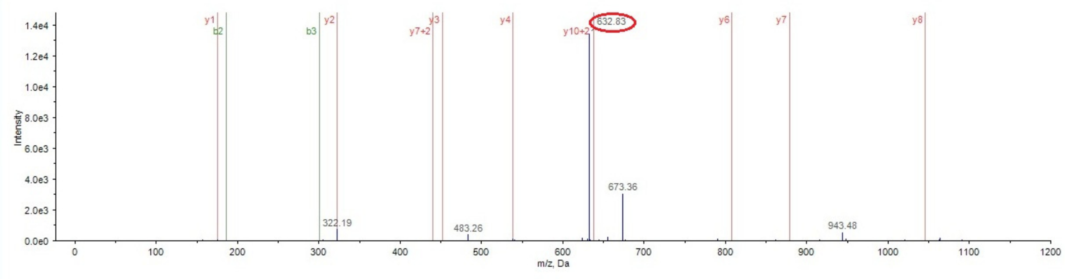

Modification: phosphorylation at S residue

GKVTpVPYAAYNLLR

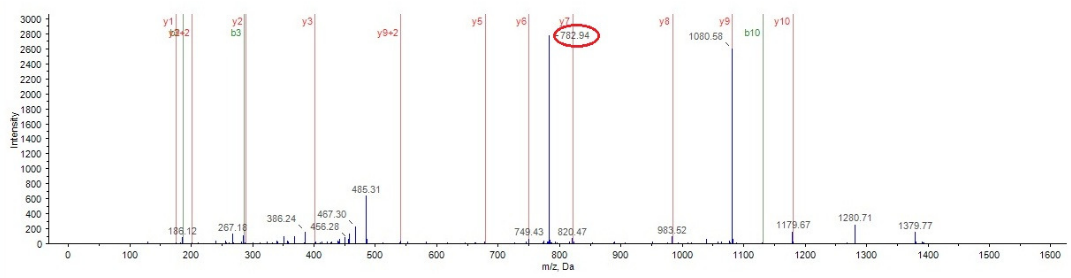

Modification: phosphorylation at T residue
